# Trail (TNF-related apoptosis-inducing ligand) induces an inflammatory response in human adipocytes

**DOI:** 10.1038/s41598-017-05932-7

**Published:** 2017-07-18

**Authors:** Verena Zoller, Jan-Bernd Funcke, Julian Roos, Meike Dahlhaus, Muad Abd El Hay, Karlheinz Holzmann, Ralf Marienfeld, Thomas Kietzmann, Klaus-Michael Debatin, Martin Wabitsch, Pamela Fischer-Posovszky

**Affiliations:** 1 Division of Pediatric Endocrinology and Diabetes, Department of Pediatric and Adolescent Medicine, University Medical Center Ulm, Ulm, Germany; 2 Core Facility Genomics, Ulm University, Ulm, Germany; 3 Institute of Pathology, Ulm University, Ulm, Germany; Department of Pediatric and Adolescent Medicine, University Medical Center Ulm, Ulm, Germany; 4 Faculty of Biochemistry and Molecular Medicine and Biocenter Oulu, University of Oulu, Oulu, Finland; 5 Department of Pediatrics and Adolescent Medicine, University Medical Center Ulm, Ulm, Germany

## Abstract

High serum concentrations of TNF-related apoptosis-inducing ligand (TRAIL), a member of the tumor necrosis factor protein family, are found in patients with increased BMI and serum lipid levels. In a model of murine obesity, both the expression of TRAIL and its receptor (TRAIL-R) is elevated in adipose tissue. Accordingly, TRAIL has been proposed as an important mediator of adipose tissue inflammation and obesity-associated diseases. The aim of this study was to investigate if TRAIL regulates inflammatory processes at the level of the adipocyte. Using human Simpson-Golabi-Behmel syndrome (SGBS) cells as a model system, we found that TRAIL induces an inflammatory response in both preadipocytes and adipocytes. It stimulates the expression of interleukin 6 (IL-6), interleukin 8 (IL-8) as well as the chemokines monocyte chemoattractant protein-1 (MCP-1) and chemokine C-C motif ligand 20 (CCL-20) in a time- and dose-dependent manner. By using small molecule inhibitors, we found that both the NFκB and the ERK1/2 pathway are crucial for mediating the effect of TRAIL. Taken together, we identified a novel pro-inflammatory function of TRAIL in human adipocytes. Our findings suggest that targeting the TRAIL/TRAIL-R system might be a useful strategy to tackle obesity-associated adipose tissue inflammation.

## Introduction

Obesity as defined by a body mass index (BMI) >30 kg/m^2^ is a disease with increasing prevalence^1, 2^. It is associated with co-morbidities such as type 2 diabetes mellitus, cardiovascular diseases and an increased cancer risk^3^. Furthermore, obesity is characterized by the excessive accumulation of triglycerides in adipose tissue, adipocyte hypertrophy, hypoxia and inflammation, which can be seen by the infiltration and accumulation of macrophages within adipose tissue^4–6^. Recent evidence suggests that members of the tumor necrosis factor (TNF) protein family contribute to adipose tissue inflammation and the development of associated co-morbidities^7, 8^. In particular, one member of the TNF superfamily, the tumor necrosis factor-related apoptosis-inducing ligand (TRAIL)^9^, was found to be increased in the serum of patients with a high BMI and serum lipid levels^10, 11^. In line with this, the expression of TRAIL was found to be increased in the adipose tissue of genetically obese (*ob/ob*) mice^12^.

TRAIL primarily acts *via* its receptors TRAIL-R1 (DR4) and TRAIL-R2 (DR5). Receptor binding leads to the recruitment of Fas-associated death domain (FADD), caspase-8 and -10 as well as cellular FLICE inhibitory protein (cFLIP) to the receptor^13, 14^. The formation of this death inducing signaling complex (DISC, primary complex) leads to the activation of the initiator caspases, *i.e*. caspase-8 or -10, by proximity-induced dimerization and subsequent self-cleavage^15^. This in turn results in the activation of effector caspases like caspase-3, -6 and/or -7 culminating in apoptosis induction^16^. Apart from this canonical pathway, TRAIL can also signal *via* a cytoplasmic complex, which is released from the DISC. In addition to FADD, cFLIP, caspase-8 and -10, this non-canonical, secondary complex consists of the receptor-interacting-protein kinase 1 (RIPK1), the adaptor protein TNF receptor type 1-associated death domain (TRADD) and the TNF receptor-associated factor 2 (TRAF2). The secondary complex is involved in the activation of kinases such as the protein kinase AKT, the classical MAP kinases extracellular signal-regulated kinases 1/2 (ERK1/2), p38 and c-Jun N-terminal kinase (JNK) as well as the nuclear factor kappa B (NFκB) pathway^17^ that can lead to transcription of anti-apoptotic and pro-proliferative genes. Indeed, TRAIL was shown to be a potent inducer of preadipocyte proliferation^18^. Furthermore, TRAIL has a significant impact on adipocyte metabolism and appears to contribute to diet-induced insulin resistance and hepatic steatosis^19^. On the molecular level, TRAIL inhibits insulin-stimulated glucose uptake and lipid formation by caspase-mediated cleavage of PPARγ^12^, hence underlining the important role of TRAIL in systemic metabolism. Interestingly, TRAIL receptor (DR5) knockout mice fed a diet high in saturated fat, cholesterol and fructose (FFC) have a reduced expression of inflammatory genes in white adipose tissue when compared to wild-type littermates^19^.

Based on the overall data, we hypothesized that TRAIL might contribute to obesity-induced adipose tissue inflammation by triggering kinase pathways that lead to cytokine and chemokine expression. However, so far it has not been investigated whether and to which extent TRAIL promotes an inflammatory response in human adipocytes. We therefore studied the impact of TRAIL on the production of inflammatory cytokines and chemokines as well as the signaling pathways underlying this effect in human preadipocytes and adipocytes.

## Results

### TRAIL induces a pro-inflammatory response in preadipocytes and adipocytes

In this study, we used the human Simpson-Golabi-Behmel syndrome (SGBS) cell strain as a model system. The cells are neither transformed nor immortalized and represent a well-characterized model system to study human adipocyte biology^20^.

**Figure 1:**
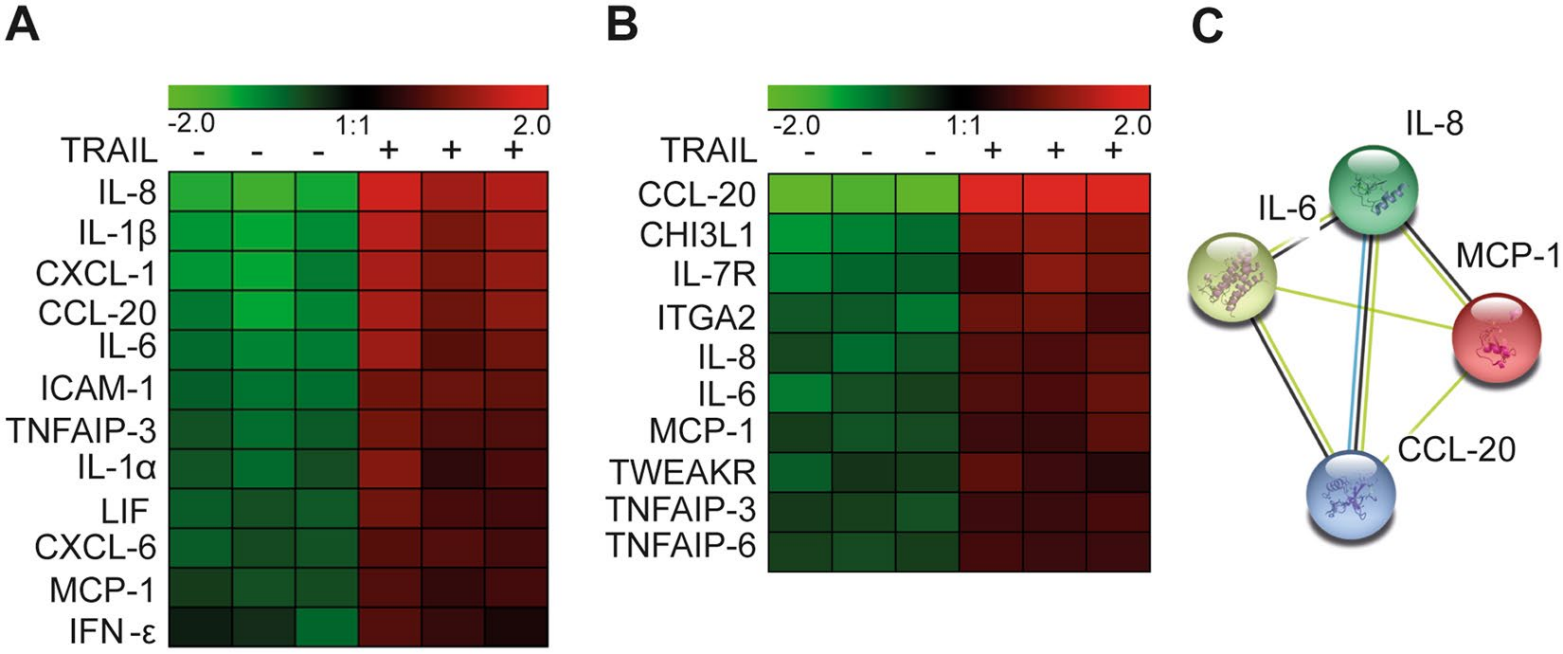
TRAIL induces a pro-inflammatory expression profile in human preadipocytes and adipocytes. SGBS preadipocytes and adipocytes on day 14 of adipogenic differentiation were treated with TRAIL (+)(30 ng/ml) or vehicle (−) as indicated. After 12 hours, RNA was harvested and subjected to mRNA array analysis (GeneChip Human Gene 1.0 ST Array; Affymetrix). Heatmaps display the TRAIL-regulated inflammatory genes in preadipocytes (**A**) and adipocytes (**B**). An evidence-based STRING 10 analysis was performed to visualize the network of TRAIL-regulated genes in preadipocytes and in adipocytes (**C**).

SGBS preadipocytes and differentiated adipocytes were treated with 30 ng/ml TRAIL. After 12 hours, RNA was isolated and subjected to an Affymetrix-based (GeneChip Human Gene 1.0 ST Array) mRNA array analysis. In SGBS preadipocytes, 38 genes showed a differential expression profile upon TRAIL treatment when compared to the control. Of these, 3 genes were down-regulated and 35 genes were up-regulated (Supplementary Table 1). Figure 1A displays the heatmap for the 12 genes differentially regulated by TRAIL in preadipocytes, which were related to inflammation (Fig. 1A). Interestingly, all inflammation-related genes regulated by TRAIL were up-regulated. In SGBS adipocytes, a total of 71 genes were regulated by TRAIL compared to the control. Of these, 36 genes were up-regulated and 35 genes were down-regulated (Supplementary Table 2). Figure 1B displays the heatmap of inflammation-related genes differentially regulated upon TRAIL treatment in adipocytes. We next performed a STRING (Search Tool for the Retrieval of Interacting Genes/Proteins) analysis to predict physical and functional protein interactions. It turned out that 5 genes were jointly up-regulated in preadipocytes as well as in adipocytes (tumor necrosis factor alpha-induced protein 3 (TNFAIP3), chemokine C-C motif ligand 20 (CCL-20), interleukin 6 (IL-6), interleukin 8 (IL-8) and monocyte chemoattractant protein 1 (MCP-1)). The 4 secreted factors are known to interact with each other (visualized in Fig. 1C).

In addition to the jointly up-regulated factors, TRAIL induced other cytokines and chemokines, *e.g*. interferon-γ (IFNγ), chemokine C-X-C motif ligand 1 (CXCL-1), chemokine C-X-C motif ligand 6 (CXCL-6), interleukin 1α (IL-1α) and interleukin 1β (IL-1β) specifically in preadipocytes. In adipocytes some cytokine receptors such as TNF-related weak inducer of apoptosis receptor (TWEAKR) and interleukin 7 receptor (IL-7R) were specifically up-regulated. Together, our expression array results indicate that TRAIL triggers a pro-inflammatory response in preadipocytes and adipocytes.

### TRAIL induces IL-6, IL-8, MCP-1 and CCL-20 production in a time- and dose-dependent manner

In order to understand the common mechanisms underlying TRAIL’s effects, our following experiments focused on those secreted factors, which were jointly up-regulated in both preadipocytes and adipocytes and studies were performed with adipocytes.

**Figure 2:**
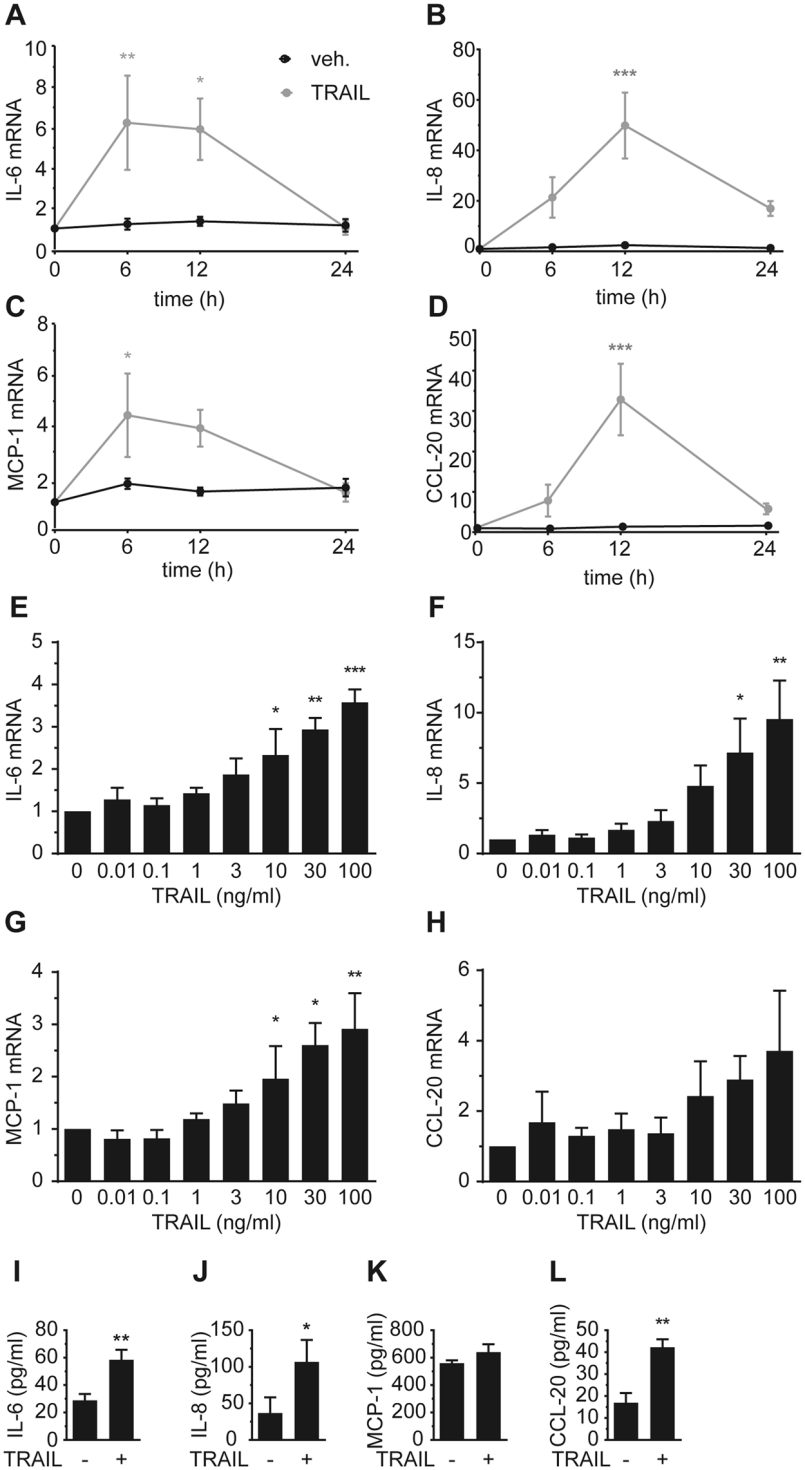
TRAIL regulates the production of cytokines and chemokines in a time- and dose-dependent manner. (**A**–**D**) SGBS adipocytes on day 14 of adipogenic differentiation were treated with TRAIL (30 ng/ml) or vehicle for 6, 12 and 24 hours. The mRNA expression of IL-6 (**A**), IL-8 (**B**), MCP-1 (**C**) and CCL-20 (**D**) was analyzed by qPCR. The mRNA levels were normalized to HPRT. Depicted are the means and SEM of 4 independent experiments. Two-way ANOVA and Sidak’s multiple comparison were used to test for statistical significance. (**E**–**H**) SGBS adipocytes on day 14 of adipogenic differentiation were treated with increasing doses of TRAIL or vehicle for 6 hours. The mRNA expression of IL-6 (**E**), IL-8 (**F**), MCP-1 (**G**) and CCL-20 (**H**) was analyzed by qPCR. The mRNA levels were normalized to HPRT. Depicted are the means and SEM of 3 independent experiments. One-way ANOVA and Dunnett’s multiple comparison were used to test for statistical significance. (**I**–**L**) SGBS adipocytes on day 14 of adipogenic differentiation were treated with TRAIL (30 ng/ml) or vehicle. After 24 hours, media supernatants were collected and IL-6 (**I**), IL-8 (**J**), MCP-1 (**K**) and CCL-20 (**L**) concentrations were determined by ELISA. Means and SEM of 3–7 independent experiments are shown. Unpaired Student’s t-tests were used to test for statistical significance. *p < 0.05, **p < 0.01, ***p < 0.001.

To identify the kinetics of cytokine and chemokine expression and to validate the array data we incubated SGBS adipocytes with 30 ng/ml TRAIL for different periods of time (6, 12 and 24 hours). Overall, TRAIL induced a transient increase of IL-6, IL-8, MCP-1 and CCL-20 mRNA expression. For IL-6 and MCP-1, maximal levels of about 6-fold and 4-fold were reached between 6 and 12 hours after TRAIL treatment, respectively (Fig. 2A and C). IL-8 and CCL-20 were maximally induced by about 50-fold and 30-fold 12 hours after TRAIL treatment, respectively (Fig. 2B and D). Thereafter, the expression levels of all these factors declined again.

In addition, TRAIL increased the mRNA expression of IL-6, IL-8, MCP-1 and CCL-20 in a dose-dependent manner with the most potent effect seen at a concentration of 100 ng/ml TRAIL (Fig. 2E–H). Importantly, ELISA measurements revealed that this induction of mRNA also resulted in protein production; TRAIL significantly enhanced the secretion of IL-6, IL-8 and CCL-20 after 24 hours of treatment (Fig. 2I–L). For MCP-1, we could not detect a significant increase, which was likely due to the already high secretion of MCP-1 (>500 pg/ml) in control cells.

### TRAIL induces IL-6, IL-8 and CCL-20 expression in human primary adipocytes

**Figure 3:**
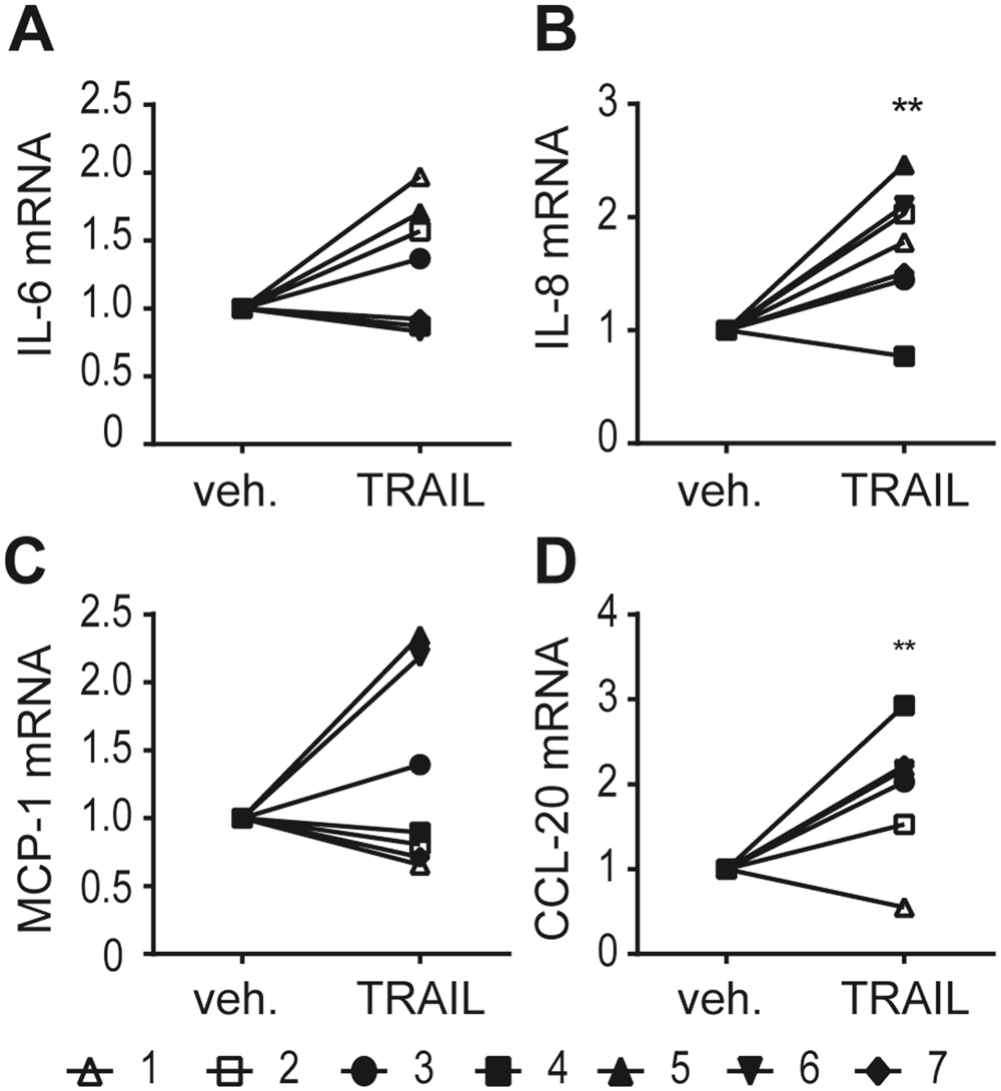
TRAIL induces the expression of cytokines and chemokines in human primary adipocytes. Human primary adipose stromal cells were isolated from white adipose tissue (n = 7) and adipogenesis was induced *ex vivo*. On day 14 of adipogenesis, the cells were treated with TRAIL (30 ng/ml) or vehicle for 6 hours and the gene expression of IL-6 (**A**), IL-8 (**B**), MCP-1 (**C**) and CCL-20 (**D**) was analyzed by qPCR. The mRNA levels were normalized to HPRT. Values are means and SEM of 7 independent experiments. Unpaired Student’s t-test was used to test for statistical significance. *p < 0.05, **p < 0.01.

To exclude cell strain-specific effects of SGBS cells we also studied human primary adipocytes, which were differentiated *in vitro* from adipose stromal cells isolated from subcutaneous adipose tissue. Upon TRAIL treatment, we observed a significant up-regulation of IL-8 and CCL-20 mRNA expression (Fig. 3A and D). Of note, there was a high inter-patient variability. Out of 7 samples, 4 displayed an up-regulation of IL-6 expression upon TRAIL stimulation (Fig. 3A). The analysis of MCP-1 mRNA expression data (Fig. 3C) displayed no consistent tendency upon TRAIL treatment, 2 samples showed a clear up-regulation, 1 sample a weak up-regulation and 4 samples remained equal or showed a tendency towards a weak down-regulation.

### Caspases are not involved in mediating the effect of TRAIL on cytokine expression

**Figure 4:**
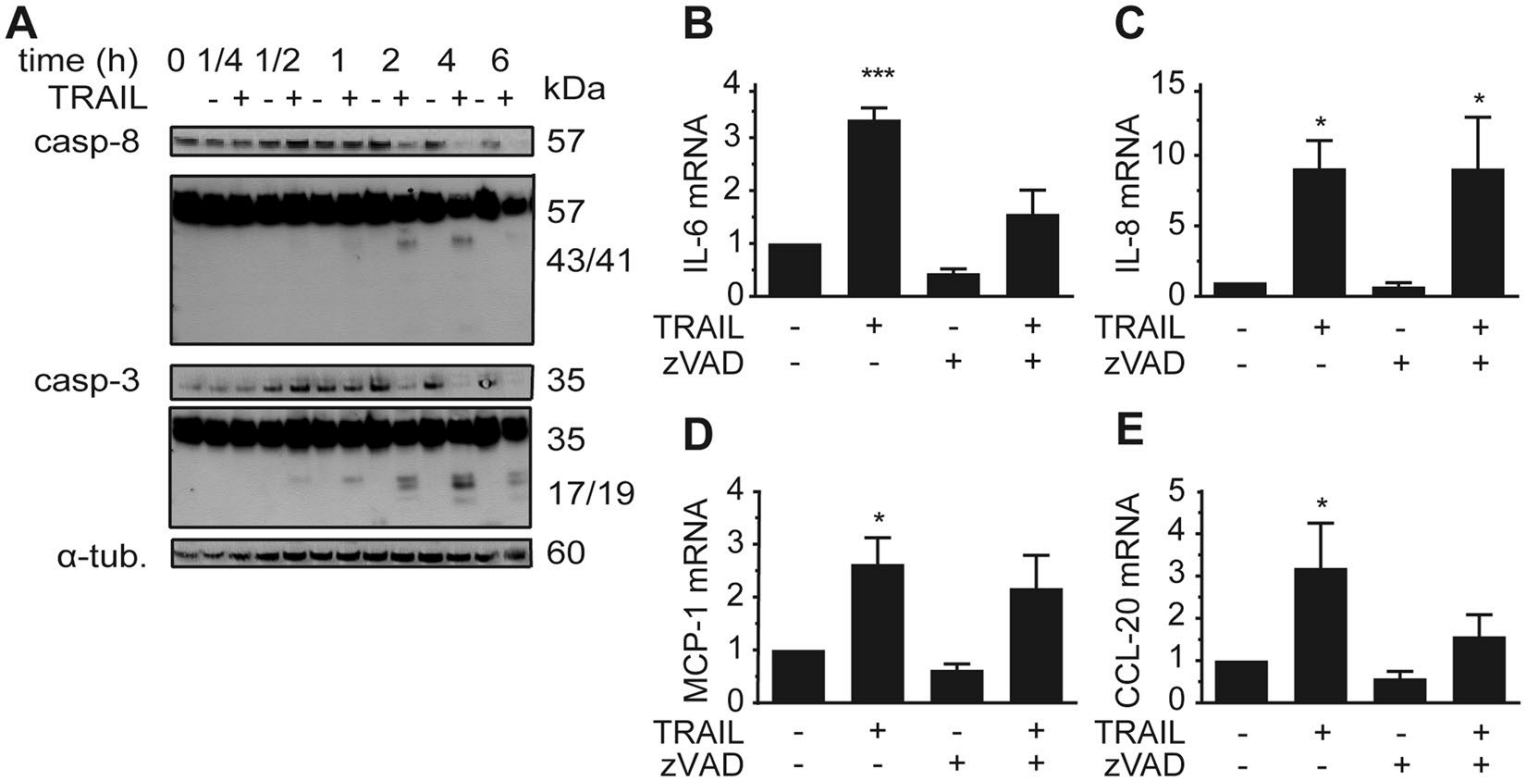
TRAIL triggers caspase activation. (**A**) SGBS adipocytes on day 14 of adipogenic differentiation were treated with TRAIL (30 ng/ml) or vehicle and protein was isolated at different timepoints (1/4, 1/2, 1, 2 and 6 hours). Cleavage of caspase-8 and caspase-3 was analyzed by Western blot. α-tubulin was used as a loading control. One representative blot out of three performed experiments is presented. (**B–E**) SGBS adipocytes on day 14 of adipogenic differentiation were treated with TRAIL (30 ng/ml) or vehicle in the absence or presence of the pan-caspase inhibitor zVAD.fmk (20 μM). After 6 hours, IL-6 (**B**), IL-8 (**C**), MCP-1 (**D**) and CCL-20 (**E**) expression was analyzed by qPCR. The mRNA levels were normalized to HPRT. Depicted are the means and SEM of 3 independent experiments. One-way ANOVA and Dunnett’s multiple comparison were used to test for statistical significance. *p < 0.05, **p < 0.01, ***p < 0.001.

Next, we sought to identify the molecular events causing the TRAIL-mediated pro-inflammatory gene expression. In a first attempt, we focused on the canonical, usually apoptosis-related pathway that can be activated by TRAIL. TRAIL stimulation in adipocytes led to a rapid cleavage of caspase-8 and caspase-3 (Fig. 4A). In line with previous results^12, 21^ we did not observe any induction of cell death. We next treated the cells with the pan-caspase inhibitor zVAD.fmk at 20 μM, a concentration known to block the TRAIL-induced formation of active caspase-8 and caspase-3 fragments^18, 22^. The TRAIL-mediated up-regulation of IL-8 and MCP-1 was not affected by caspase inhibition (Fig. 4C and D), whereas the up-regulation of IL-6 and CCL-20 seemed to be partially inhibited (Fig. 4B and E). Interestingly, caspase inhibition alone resulted in a down-regulation of IL-6 and CCL-20 mRNA suggesting that the expression of those factors requires some basal caspase activity. The fold-induction of cytokine and chemokine mRNA expression by TRAIL treatment was similar between zVAD.fmk and vehicle-treated cultures. Therefore, we concluded that the observed caspase activation is not responsible for the effects of TRAIL on inflammatory gene expression.

### TRAIL induces a phosphorylation of IκBα

We next asked whether the nuclear factor kappa B (NFκB) pathway might be responsible for TRAIL’s effects on inflammatory gene expression. This was based on recent findings indicating that IL-6, IL-8 and MCP-1 are all NFκB targets^23, 24^ and that TRAIL can induce the NFκB activation in other cell types^25, 26^. We therefore treated SGBS adipocytes with TRAIL and analyzed the phosphorylation of a central NFκB upstream regulator, the inhibitor of NFκB alpha (IκBα) protein. The phosphorylation of IκBα induces its degradation, thus activating the NFκB pathway.

**Figure 5:**
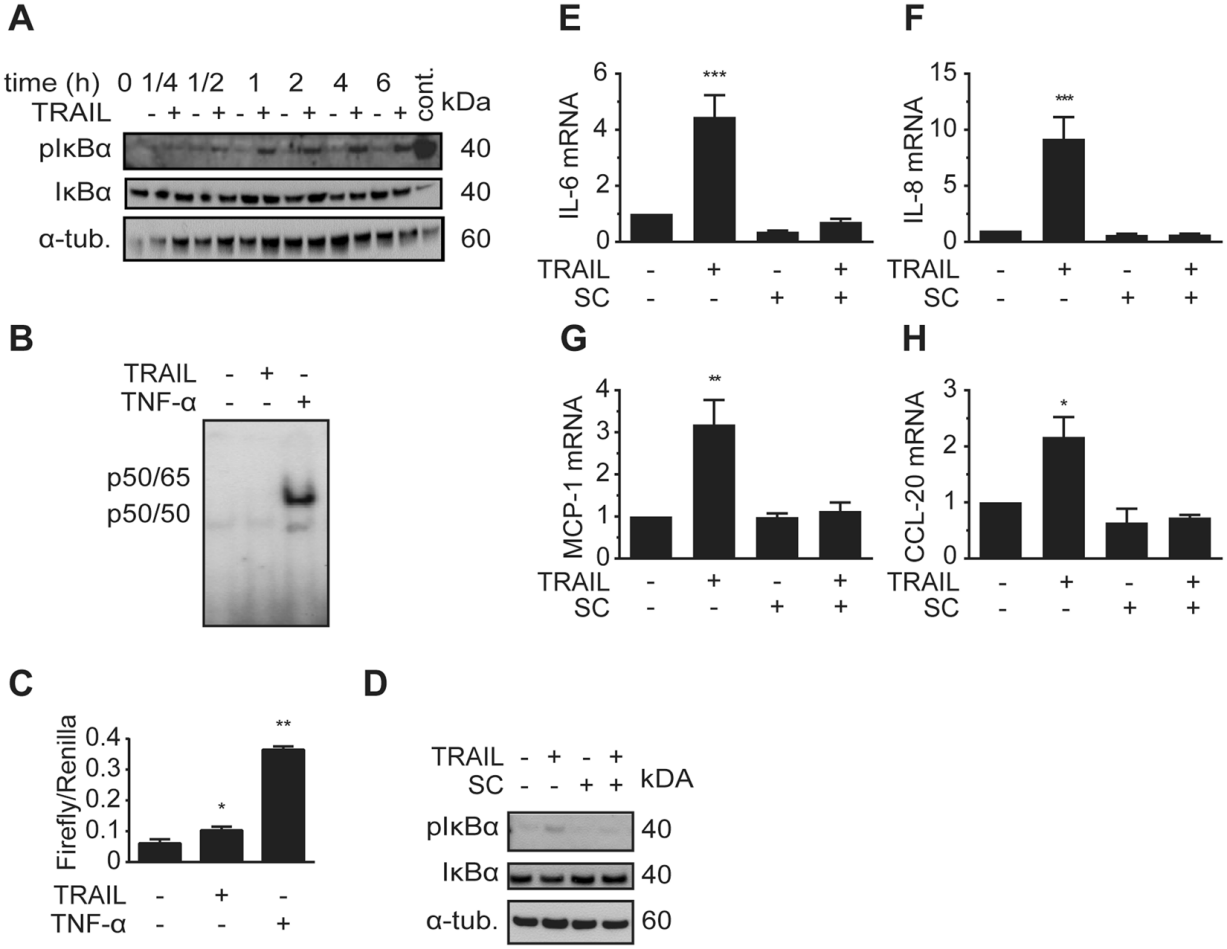
TRAIL induces the phosphorylation of IκBα. (**A**) SGBS adipocytes on day 14 of adipogenic differentiation were treated with TRAIL (30 ng/ml) or vehicle and protein was isolated at different timepoints (1/4, 1/2, 1, 2 and 6 hours). Cells stimulated with macrophage-conditioned medium (MaCM) were used as a positive control. The phosphorylation of IκBα was analyzed by Western blot. α-tubulin was used as a loading control. One representative blot out of three performed experiments is presented. (**B**) SGBS adipocytes on day 14 of adipogenic differentiation were treated for 2 hours with TRAIL (30 ng/ml), TNF-α (30 mg/ml) or vehicle and nuclear extracts were prepared. DNA binding activity of NFκB was analyzed by electrophoretic mobility shift assay (EMSA). One representative experiment out of three performed experiments is presented. (**C**) SGBS adipocytes on day 7 of adipogenic differentiation were transfected with NFκB Firefly luciferase reporter vector and Renilla luciferase control reporter vector. On day 9, cells were treated for 24 hours with TRAIL (30 ng/ml), TNF-α (30 mg/ml) or vehicle and luciferase activity was determined. Values are means and SEM of 3 different experiments. Unpaired Student´s t-test was used to test for statistical significance. (**D-H**) SGBS adipocytes on day 14 of adipogenic differentiation were treated with TRAIL (30 ng/ml) or vehicle in the absence or presence of the IKK inhibitor SC-514 (100 μM). After 6 hours, the phosphorylation of IκBα was analyzed by Western blot (**D**). α-tubulin was used as a loading control. One representative blot out of three performed experiments is presented. Also, the expression of IL-6 (**E**), IL-8 (**F**), MCP-1 (**G**) and CCL-20 (**H**) was assessed by qPCR. The mRNA levels were normalized to HPRT. Depicted are the means and SEM of 4 independent experiments. One-way ANOVA and Dunnett’s multiple comparison were used to test for statistical significance. *p < 0.05, **p < 0.01, ***p < 0.001.

Treatment of adipocytes with TRAIL resulted in a phosphorylation and very weak degradation of IκBα after 6 hours (Fig. 5A and Supplementary Figure S1 for Image J analysis) suggesting mild NFκB activation.

We then decided to perform an electrophoretic mobility shift assay (EMSA) to assess NFκB DNA binding activity in our cells. While nuclear extracts from adipocytes treated with TNF-α, a known inducer of NFκB in SGBS cells, displayed a clearly visible signal on the gel, representing NFκB DNA binding activity, there was no increase in signal in nuclear extracts obtained from TRAIL-treated adipocytes (Fig. 5B). Since the IκBα Western blots had shown a phosphorylation and weak degradation of IκBα, we concluded that the EMSA might not be sensitive enough to detect a mild activation of NFκB taking place.

In order to verify the activation of NFκB we decided to apply a reporter gene assay to measure NFκB transcriptional activity. We found that TRAIL significantly enhanced luciferase activity, although this was much lower than the activity gained upon TNF-α addition (Fig. 5C). To further clarify an involvement of NFκB, we used the small molecule inhibitor SC-514 (100 μM) to inhibit NFκB activation. SC-514 blocks the signal upstream of IκBα, the protein kinase IκB kinase-β (IKK-β). The TRAIL-induced phosphorylation of IκBα was effectively blocked by SC-514 (Fig. 5D), demonstrating that this compound is indeed suitable for blocking the canonical NFκB pathway. The addition of SC-514 abolished the effect of TRAIL on IL-6, IL-8, MCP-1 and CCL-20 mRNA expression (Fig. 5E–H). Also BAY 11-7082, a well-characterized inhibitor of cytokine-induced IκBα phosphorylation displayed a comparable effect (Supplementary Figure S2). We furthermore performed siRNA-mediated knockdowns of IKK-α and IKK-β to curb NFκB function. Unfortunately, we did not achieve a successful knockdown of both IKK forms in adipocytes (data not shown). However, in preadipocytes we were able to achieve a satisfactory knockdown (Supplementary Figure S3) that partially inhibited the TRAIL-induced up-regulation of the studied chemokines and cytokines (Supplementary Figure S3). All in all, these data demonstrate that the canonical NFκB pathway participates in the TRAIL-induced up-regulation of IL-6, IL-8, CCL-20 and MCP-1 expression.

### TRAIL induces a phosphorylation of ERK1/2

**Figure 6:**
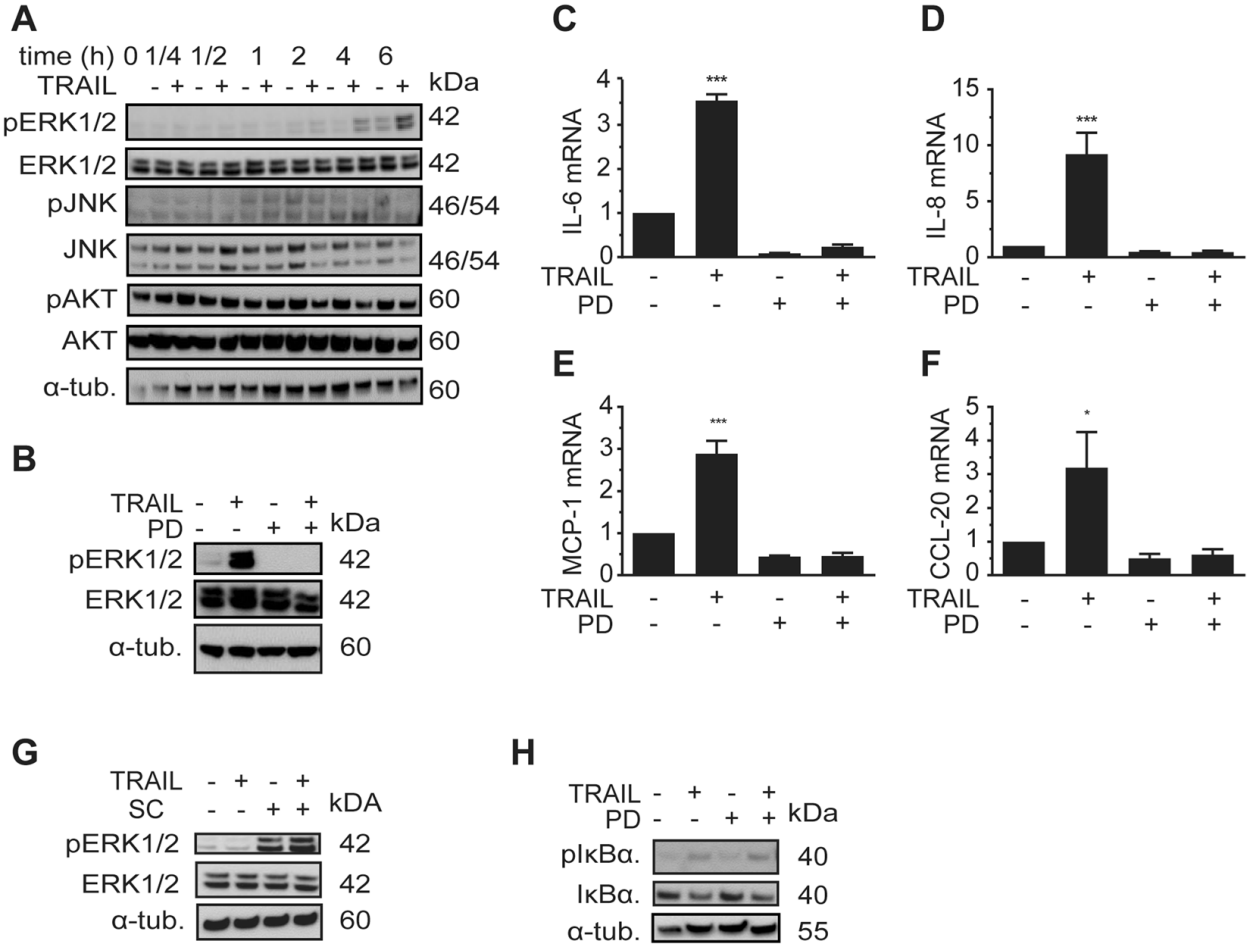
TRAIL induces the phosphorylation of ERK1/2. (**A**) SGBS adipocytes on day 14 of adipogenic differentiation were treated with TRAIL (30 ng/ml) or vehicle and protein was isolated at different timepoints (1/4, 1/2, 1, 2 and 6 hours). The phosphorylation of ERK1/2, JNK and AKT was determined by Western blot. (**B**–**F**) SGBS adipocytes on day 14 of adipogenic differentiation were treated with TRAIL (30 ng/ml) or vehicle in the absence or presence of the MEK1/2 inhibitor PD-0325901 (100 nM). After 6 hours, the phosphorylation of ERK1/2 and IκBα was analyzed by Western blot (**B**). α-tubulin was used as a loading control. One representative blot out of three performed experiments is presented. Also, IL-6 (**C**), IL-8 (**D**), MCP-1 (**E**) and CCL-20 (**F**) was analyzed by qPCR. The mRNA levels were normalized to HPRT. Depicted are the means and SEM of 4 independent experiments. One-way ANOVA and Dunnett’s multiple comparison were used to test for statistical significance. *p < 0.05, **p < 0.01, ***p < 0.001.

TRAIL is able to activate kinases such as AKT, ERK1/2 and JNK *via* a non-canonical signaling route. To address this possibility, we treated SGBS adipocytes with TRAIL and analyzed for phosphorylation of the above mentioned kinases at different time points. After 4 and 6 hours, we observed a TRAIL-dependent phosphorylation of ERK1/2 in adipocytes while the kinases AKT and JNK were not activated (Fig. 6A). To elucidate an involvement of ERK1/2 in mediating TRAIL’s effects, we used the small molecule inhibitor PD-0325901 (100 nM) to specifically block the MAPK/ERK kinases 1/2 (MEK1/2), the specific upstream kinases of ERK1/2. PD-0325901 completely abolished the TRAIL-induced phosphorylation of ERK1/2 (Fig. 6B). Importantly, inhibition of the ERK1/2 pathway also abolished the TRAIL-induced up-regulation of IL-6, IL-8, MCP1 and CCL-20 mRNA expression (Fig. 6C–F). These data demonstrate that the ERK1/2 pathway is a key player in the TRAIL-induced cytokine and chemokine expression.

Finally, we wanted to know whether TRAIL interlinks the ERK1/2 and the NFκB pathways. Interestingly, inhibition of the ERK1/2 pathway by PD-0325901 did not interfere with TRAIL-mediated IκBα phosphorylation (Fig. 6H). *Vice versa*, inhibition of the NFκB pathway by SC-514 did not interfere with ERK1/2 phosphorylation (Fig. 6G). Together, these data indicate that in adipocytes TRAIL activates the ERK1/2 and NFκB pathways concurrently and without significant interactions.

Overall, we have demonstrated that TRAIL induces a pro-inflammatory response in human adipocytes in an ERK1/2- and NFκB-dependent manner.

## Discussion

Adipose tissue is an important endocrine organ that is in permanent crosstalk with other organ systems of the body^27^. The inflammatory state of adipose tissue in obesity is supposed to play a causal role in the pathogenesis of its comorbidities^3, 28, 29^. The current study unraveled two entirely new aspects with respect to the inflammatory process in adipose tissue. First, our data demonstrate for the first time that TRAIL induces the production of pro-inflammatory cytokines and chemokines in human preadipocytes and adipocytes. Our microarray analyses revealed four TRAIL-regulated inflammation-related secreted factors that were shared by both preadipocytes and adipocytes. Second, by showing that this induction of cytokine and chemokine expression occurs in an ERK1/2- and NFκB-dependent manner, the current study unveiled the underlying mechanisms by which TRAIL can induce an inflammatory response in human adipocytes.

To our current knowledge, the effect of TRAIL on the secretion profile of adipocytes and preadipocytes has not been investigated yet. TNF-α, another member of the TNF superfamily, is known to have an impact on the secretion products of adipose tissue. TNF-α also stimulates the production of pro-inflammatory cytokines and chemokines such as IL-6, IL-8 and MCP-1 in adipocytes^30, 31^. The induction of these cytokines and chemokines by TNF-α is mediated by a strong activation of the NFκB pathway alone^32, 33^. Furthermore, TNF-α knockout mice are protected against obesity-induced insulin resistance^7^. For TRAIL and with respect to adipose tissue, the mediating pathways were not clear yet. However, in other cell types such as glioblastoma cells, NFκB but also MAP kinases such as ERK1/2, p38 and JNK as well as canonical TRAIL signaling involving caspase activation were shown to be responsible for TRAIL’s effects on cytokine and chemokine production^17, 25, 34, 35^. Our experiments clearly demonstrate that TRAIL induces a cleavage of caspase-8 and caspase-3 under the chosen conditions. While the activation of caspases is a prerequisite for TRAIL’s effects on adipocyte metabolism and adipogenic differentiation, the current data indicate that caspase cleavage does not seem to be involved in mediating its effects on cytokine and chemokine expression^12, 22^. This is in line with earlier studies showing that TRAIL is a potent mitogen in human preadipocytes and that even though a robust cleavage of caspase-8 and caspase-3 was observed, this proliferative effect was similarly independent of caspase activity^18^.

We therefore propose that TRAIL activates several pathways at the same time, but the single activated pathways are responsible for very specific functions within the cell. For example, caspases seem to play a role for adipocyte differentiation and metabolic regulation involving PPARγ^12, 22^, whereas activation of ERK1/2 appears to be crucial for preadipocyte proliferation^18^. However, we also show here that a simultaneous engagement of both the canonical NFκB and the ERK1/2 pathway mediate the effect of TRAIL on cytokine and chemokine production. Both pathways were previously described as important regulators of cytokine and chemokine production^23, 24, 36^. It was reported that IKK-β is able to activate ERK1/2^37^ and the other way round that ERK1/2 is able to activate NFκB^38, 39^. Furthermore, in vascular smooth muscle cells it was reported that activation of ERK1/2 is required for a persistent activation of the NFκB pathway by degradation of IκBβ^40^. The current study did not hint at a crosstalk between the NFκB and ERK1/2 pathways in adipocytes as our data obtained with small molecule inhibitors of both pathways did not show any interconnection. However, an interconnection can also not be fully ruled out since inhibition of the ERK1/2 as well as the NFκB axis completely inhibited the effect of TRAIL. If both pathways would simply run in parallel in response to a common upstream activator, we would rather expect that there is only a partial effect, *e.g*. the TRAIL-induced NFκB route should still be active upon ERK1/2 inhibition. However, this was not the case and by taking this into account it appears more likely that both pathways end up in the activation of a common downstream signal. Its identification requires further investigations and is behind the scope of this study.

Since the discovery of TRAIL and its receptors, their function has been mostly studied in the context of malignant and inflammatory diseases^41, 42^. A potential role in obesity and metabolic diseases has just recently been proposed, but previous findings have been rather conflicting. On the one hand, genetically obese mice showed an increased expression of the mouse TRAIL receptor DR5 in adipose tissue suggesting a role in adipose tissue function^12^. In support of this, DR5^−/−^ mice fed an FFC diet are protected from weight gain, insulin resistance and hepatic steatosis^19^. Furthermore, DR5^−/−^ mice had lower adipose tissue inflammation on an FFC diet. The mRNA levels of MCP-1 and TNF-α were significantly reduced in knockout animals compared to wild-type littermates supporting a pro-inflammatory role of DR5^19^.

On the other hand, when mice deficient of both TRAIL and apolipoprotein E (TRAIL^−/−^ ApoE^−/−^) were fed a high-fat diet, the development of diabetic features, such as increased weight and impaired glucose tolerance were observed^43^. The knockout animals also had higher numbers of CD11b^+^ leukocytes and higher levels of IL-6 in the circulation^43^. In line with these findings is a study showing that in mice fed a high-fat diet, weekly injections of recombinant TRAIL resulted in reduced weight gain and improved hyperglycemia as well as hyperinsulinemia^44^. Furthermore, it caused not only a reduction of IL-6 and TNFα on mRNA expression in white adipose tissue, but also decreased circulating IL-6 and TNFα^44^. Our findings suggest that an anti-inflammatory action of TRAIL is probably not mediated at the level of the adipocyte. However, our findings are solely based on *in vitro* experiments, which are limited since the crosstalk with other cell and organ systems is not taken into account. Our results support a pro-inflammatory role for TRAIL in human adipocytes. TRAIL stimulated the secretion of several pro-inflammatory factors such as IL-6, IL-8 and MCP-1, which were previously shown to be associated with obesity and insulin resistance^45, 46^. CCL-20 has been described as an adipokine and its expression in adipocytes increases with elevated BMI^47^.

All these factors might act locally in an auto- and/or paracrine manner and further fuel the inflammatory process within adipose tissue, but they might also spill over into the circulation and thus impact other organ systems. For example, IL-6 is known to induce insulin resistance in skeletal muscle and liver cells^48, 49^. The importance of our findings with respect to the pro-inflammatory role of TRAIL and its receptor in adipose tissue is further underlined by a study in mice with an adipocyte-specific knockout of CD95, a related member of the TNF receptor superfamily. When these mice were fed with a high-fat diet, they did not display glucose intolerance, adipose tissue inflammation and liver steatosis the way their wild-type littermates did^8^.

The reasons underlying this partially conflicting data are currently not known. Different genetic models, diet-related and tissue-specific as well as species-specific effects may, alone or in combination, account for it. Despite any conflict, all current studies underline that the TNF receptor superfamily and their ligands have an impact on adipose tissue homeostasis. This is of particular importance because recombinant TRAIL or TRAIL receptor agonists are currently used in clinical trials for the treatment of cancer. In one phase I trial a maximum serum concentration of 259 μg/ml TRAIL was reached, which is way above the highest concentrations used in this study^50^. Although no severe side effects of TRAIL treatment except fatigue, nausea, vomiting, fever, anemia and constipation^50^ have been reported yet, the possible pro-inflammatory effect on adipose tissue should be taken into account.

Altogether, our study adds a new piece of information to the biological functions of TRAIL. We describe a novel pro-inflammatory function of TRAIL in human adipocytes, which is mediated by ERK1/2 and NFκB. Based on our findings, blocking the function of TRAIL in adipose tissue might be beneficial to either prevent or ameliorate adipose tissue inflammation.

## Materials and Methods

### Recombinant proteins, small molecule inhibitors and cell culture material

Recombinant human TRAIL (375-TEC) was purchased from Bio-Techne (Wiesbaden-Nordenstadt, Germany), TNF-α from Merck (Darmstadt, Germany), zVAD.fmk from Bachem (Bubendorf, Switzerland), SC-514 from Bio-Techne (Wiesbaden-Nordenstadt, Germany), PD-0325901 from Selleckchem (Houston, Texas, USA) and Bay 11-7082 from Invivogen (San Diego, California, USA). Cell culture media and buffers were from Thermo Fisher Scientific (Darmstadt, Germany).

### Ethical Note

All procedures were performed according to the Declaration of Helsinki guidelines and authorized by the ethics committee of Ulm University (entry number 368/13). Written informed consent was obtained from all subjects in advance.

### Subjects and primary human adipose stromal cell isolation

Primary human adipose stromal cells were isolated from subcutaneous white adipose tissue obtained from seven subjects undergoing plastic surgery. Collagenase (Sigma-Aldrich, Munich, Germany) digestion was performed as described elsewhere^50^. The mean age was 31.2 ± 12.3 years, the mean BMI was 24.9 ± 5.2 kg/m^2^. Cells were subjected to adipogenic differentiation and treated with TRAIL as described for SGBS cells.

### Cell culture

Human Simpson-Golabi-Behmel syndrome (SGBS) cells were used as a model system of adipocyte biology^20^. Cells were passaged in DMEM/F-12 (1:1), 100 U/ml penicillin, 100 μg/ml streptomycin (Life Technologies, Darmstadt, Germany), 17 μM D-pantothenic acid and 33 μM biotin (Sigma-Aldrich, Munich, Germany) (referred to as basal medium) supplemented with 10% fetal bovine serum. For the induction of adipogenic differentiation, subconfluent cell cultures were washed with PBS and adipogenic medium (serum-free basal medium with 20 nM human recombinant insulin, 100 nM cortisol, 200 pM triiodothyronine and 10 μg/ml transferrin) supplemented with 2 μM rosiglitazone, 25 nM dexamethasone and 250 μM isobutylmethylxanthine was added. After four days, the medium was changed to adipogenic basal medium alone. On day 14 of adipogenesis TRAIL or an equal amount of vehicle (PBS + 0.01% bovine serum albumin) was added directly to the media.

### RNA isolation and cDNA preparation

Isolation of total RNA was performed using the peqGOLD HP total RNA kit (peqlab, Erlangen, Germany) or the Direct-zol RNA Mini Prep kit (Zymo Research Corporation, Irvine, California, USA) according to the manufacturers’ instructions. cDNA synthesis was conducted using SuperScript II Reverse Transcriptase (Thermo Fisher Scientific, Darmstadt, Germany) according to the manufacturer’s instructions.

### mRNA Expression Arrays

Subconfluent cultures of SGBS preadipocytes were treated with TRAIL or an equal amount of vehicle in basal medium supplemented with 10% fetal bovine serum. Adipocytes were treated with TRAIL or an equal amount of vehicle as described above. RNA was isolated after 12 hours. Triplicate experiments were performed. Integrity of the RNA was analyzed using a Bionanalyzer (Agilent, Santa Clara, California, USA). The RNA array analysis was performed by the Core Facility Genomics University Medical Center Ulm.

In brief, 200 ng total RNA as starting material and 5.5 μg ssDNA were used per hybridization (GeneChip Fluidics Station 450; Affymetrix, Santa Clara, California, USA). The total RNAs were amplified and labeled following the Whole Transcript (WT) Sense Target Labeling Assay (http://www.affymetrix.com). Labeled ssDNA was hybridized to Human Gene 1.0 ST Affymetrix GeneChip arrays. The chips were scanned with a Affymetrix GeneChip Scanner 3000 and subsequent images analyzed using Affymetrix Expression Console Software.

A transcriptome analyses was performed using BRB-ArrayTools developed by Dr. Richard Simon and BRB-ArrayTools Development Team (http://linus.nci.nih.gov/BRB-ArrayTools.html). Raw feature data were normalized and log_2_ intensity expression summary values for each probe set were calculated using robust multiarray average^51^.

#### Filtering

Genes showing minimal variation across the set of arrays were excluded from the analysis. Genes whose expression differed by at least 1.5 fold from the median in at least 20% of the arrays were retained.

#### Class comparison

We identified genes that were differentially expressed among the two classes using a two sample t-test. Genes were considered statistically significant if their p value was less than 0.05 and displayed a fold change between the two groups of at least 1.5 fold.

We used the Benjamini and Hochberg correction to provide 90% confidence that the false discovery rate was less than 10%^52^. The false discovery rate is the proportion of the list of genes claimed to be differentially expressed that are false positives. Functional protein association networks between mutually differentially expressed genes were identified using the STRING 10 program (http://string-db.org/).

### Quantitative real-time PCR

qPCR was performed on a LightCycler 2.0 instrument using the My-Budget 5x EvaGreen qPCR Mix (Bio-Budget, Krefeld, Germany). The mRNA levels of the genes of interest were first normalized to HPRT (hypoxanthine-guanine-phosphoribosyl transferase, ΔCT value) and then to the corresponding control condition (ΔΔCT value)^53^. The following oligonucleotide primers were obtained from Thermo Scientific (Ulm, Germany): HPRT forw 5′-GAG ATG GGA GGC CAT CAC ATT GTA GCC CTC-3′, HPRT rev 5′-CTC CAC CAA TTA CTT TTA TGT CCC CTG TTG ACT GGT C-3′; IL-6 forw 5′-TAC CCC CAG GAG AAG ATT CC-3′, IL-6 rev 5′-TTT TCT GCC AGT GCC TCT TT-3′; IL-8 forw 5′-TGC CAA GGA GTG CTA AAG AAC TTA GAT GTC AG-3′, IL-8 rev 5′-AGC TTT ACA ATA ATT TCT GTG TTG GCG CAG TG-3′; MCP-1 forw 5′-TCC CAA AGA AGC TGT GAT CTT CAA GAC C-3′, MCP-1 rev 5′-AGT GAG TGT TCA AGT CTT CGG AGT TTG G-3′; CCL-20 forw 5′-GCG GCG AAT CAG AAG CAA GCA ACT TTG AC-3′, CCL-20 rev 5′-GCA TTG ATG TCA CAG CCT TCA TTG GCC AG-3′.

### Protein extraction and Western blot

Whole protein extracts were obtained by washing the cells with ice cold PBS and adding lysis buffer (10 mM Tris-HCl pH 7.5, 150 mM NaCl, 2 mM EDTA, 1% Triton X-100, 10% glycerol) supplemented with 1X cOmplete Proteinase Inhibitor Cocktail and 1X PhosSTOP Phosphatase Inhibitor Cocktail (Roche Diagnostics, Mannheim, Germany). Cells were detached by scraping. The lysates were incubated for 20 minutes at 4 °C and afterwards centrifuged at 14000 rpm for 30 minutes at 4 °C. Western blot analysis was performed as described elsewhere^18^. The following antibodies were used: rabbit anti-phospho AKT (S473), rabbit anti-AKT, mouse anti-phospho ERK1/2 (T202/Y204), rabbit anti-phopsho JNK (T183/Y185), mouse anti-phospho IκBα (S32/S36), rabbit anti-IκBα, rabbit anti-caspase-3 (Cell signaling, Cambridge, UK), mouse anti-JNK (BD, Heidelberg, Germany), mouse anti-caspase-8 (Alexis, Grünberg, Germany), rabbit anti-ERK1/2, mouse anti-β-actin (Sigma-Aldrich, Munich, Germany), mouse anti-caspase-8 (Enzo LifeSciences, Lörrach, Germany) and mouse anti-α-tubulin (Calbiochem/EMD Millipore, Darmstadt, Germany). HRP-conjugated goat anti-mouse IgG and goat anti-rabbit IgG were from Santa Cruz Biotechnology (Heidelberg, Germany). Full length blots are provided in the Supplementary Appendix.

### ELISA

SGBS adipocytes were treated with 30 ng/ml TRAIL as described above. Media supernatants were harvested after 24 hours and cleared by centrifugation. The ELISA for IL-6, IL-8 and MCP-1 was implemented according to the manufacturer’s instructions using Ready-SET-Go! ELISA kits (eBioscience, Vienna, Austria). The ELISA for CCL-20 was performed using the CCL20/MIP-3 alpha ELISA kit from Novus Biologicals (Littleton, Colorado, USA) following the manufacturer’s instructions. Absorbance was measured on a microplate spectrophotometer reader (ELx800 Absorbance Microplate Reader, BioTek, Bad Friedrichshall, Germany).

### Electrophoretic Mobility Shift Assay (EMSA)

SGBS adipocytes were treated for 2 hours with TRAIL (30 ng/ml), TNF-α (30 mg/ml) as a positive control or vehicle as a negative control. SGBS cells were collected from 10 cm dishes by scraping and centrifugation (10,000 g for 5 min at 4 °C). The preparation of nuclear extracts and EMSAs were performed as previously described^54^. The used oligonucleotides were purchased from Biomers.net (Ulm, Germany). The sequence of the NFκB sense and NFκB antisense oligonucleotides were 5′-AGT TGA GGG GAC TTT CCC AGG C-3′ and 5′-GCC TGG GAA AGT CCC CTC AAC T-3′, respectively. The sense oligonucleotide was labeled with γ-^32^P-ATP (Hartmann Analytics, Braunschweig, Germany) using T4-polynucleotide kinase (Thermo Fisher Scientific, Darmstadt, Germany).

### Reporter gene assay

On day 7 of differentiation SGBS adipocytes were nucleofected with a NFκB Firefly luciferase reporter vector containing five copies of an NFκB response element that drives transcription of the Firefly luciferase reporter gene and Renilla luciferase control reporter vector using the Neon Transfection System (Thermo Fisher Scientific, Darmstadt, Germany). First, adipocytes were trypsinized, counted and a cell solution of 600000 cells was centrifuged and resuspended in 105 μl Resuspension Buffer B of the Neon Transfection Kit (100 μl). The nucleofection was performed with 7.5 μg of the NFκB Firefly luciferase reporter vector^55, 56^ and with 0.75 μg of the Renilla luciferase control reporter vector (pRL-TK; Promega, Heidelberg) at 1400 Volt with three electric shocks for 10 ms each. Then, 75000 cells were seeded into one well of a 12-well plate. After 24 hours the adipocytes were treated for additional 24 hours with either TRAIL (30 ng/ml), TNF-α (30 ng/ml) as a positive control or vehicle as a negative control. The cell lysates were harvested by scraping and the luciferase activities were measured with the Dual-Glo Luciferase Reporter Kit (Promega, Madison, Wisconsin, USA) according to the manufacturer’s instructions. Luminescence was recorded in a Multimode Microplate Reader (Mithras LB 940, Berthold, Bad Wildbad, Germany).

## Electronic supplementary material

Supplementary Information
