# Supplementary material for: Trail (TNF-related apoptosis-inducing ligand) induces an inflammatory response in human adipocytes
Source: Sci Rep. 2017 Jul 18;7:5691. doi: 10.1038/s41598-017-05932-7 (PMC5515939; doi:10.1038/s41598-017-05932-7)
Supplement: Supplementary file 1 — Supplementary Information [file 41598_2017_5932_MOESM1_ESM.pdf]

Supplementary Appendix

TRAIL (TNF-related apoptosis-inducing ligand) induces an inflammatory response in human adipocytes

Verena Zoller [1], Jan-Bernd Funcke [1], Julian Roos [1], Meike Dahlhaus [1], Muad Abd El Hay [1], Karlheinz Holzmann [2], Ralf Marienfeld [3], Thomas Kietzmann [4], Klaus-Michael Debatin [5], Martin Wabitsch [1], and Pamela Fischer-Posovszky [1]

Affiliations

[1] Division of Pediatric Endocrinology and Diabetes, Department of Pediatric and Adolescent Medicine, University Medical Center Ulm, Ulm, Germany;

[2] Core Facility Genomics, Ulm University, Ulm, Germany;

[3] Institute of Pathology, Ulm University, Ulm, Germany; Department of Pediatric and Adolescent Medicine, University Medical Center Ulm, Ulm, Germany;

[4] Faculty of Biochemistry and Molecular Medicine and Biocenter Oulu, University of Oulu, Oulu, Finland;

[5] Department of Pediatric and Adolescent Medicine, University Medical Center Ulm, Ulm, Germany

Corresponding author

Pamela Fischer-Posovszky, PhD

Division of Pediatric Endocrinology and Diabetes

Department of Pediatric and Adolescent Medicine

University Medical Center Ulm

Eythstr. 24

89075 Ulm

Phone: +49 (0)731 500 57415

Email: pamela.fischer@uniklinik-ulm.de

Supplementary Table 1

SGBS preadipocytes were treated with TRAIL (30 ng/ml) or vehicle for 12 hours. RNA was harvested and subjected to mRNA array analysis (Affymetrix, GeneChip Human Gene 1.0 ST Array). This table displays the genes that were differentially regulated by TRAIL in preadipocytes. Downregulated genes are given in dark green, upregulated genes in red.

Probe Set	Gene	Fold-change TRAIL vs veh.	Parametric p-value
8148059	DEPTOR	0.45	0.0008639
8048749	KCNE4	0.48	0.0002156
8054254	AFF3	0.57	0.0024321
7964733	RPSAP52	1.89	0.0015996
8160435	IFNE	1.96	0.0090086
8015221	KRTAP4-11	2.04	0.0012079
8065353	THBD	2.08	0.000115
8160439	MIR31	2.08	0.0041807
8105267	ITGA2	2.13	0.0000562
8143127	FAM180A	2.13	0.0000897
8054344	RFX8	2.13	0.0007926
8114797	SPRY4	2.17	0.0000004
8015223	KRTAP4-12	2.17	0.000478
8019565	KRTAP4-12	2.17	0.000478
8015218	KRTAP4-7	2.17	0.0006165
8015230	KRTAP4-12	2.17	0.0007172
8021635	SERPINB2	2.22	0.0005665
8006433	CCL2	2.27	0.0001043
7928429	PLAU	2.33	0.0000703
8112045	ESM1	2.33	0.000125
8154233	CD274	2.38	0.0000764
8025402	ANGPTL4	2.38	0.0001774
8052872	TGFA	2.44	0.0000084
7951271	MMP1	2.44	0.0004807
8095688	CXCL6	2.50	0.0000193
8092726	CLDN1	2.50	0.0003813
7965335	DUSP6	2.56	0.0001134
8075310	LIF	2.56	0.0001682
8114572	HBEGF	2.63	0.0000311
8144786	SLC7A2	2.70	0.0000674
8149825	STC1	2.70	0.000361
8054712	IL1A	2.70	0.0015886
7943413	BIRC3	2.78	0.0002354
8122265	TNFAIP3	2.86	0.0001212
8025601	ICAM1	3.23	0.0000074

8131803	IL6	3.70	0.0002961
8048864	CCL20	4.55	0.0001772
8095697	CXCL1	5.00	0.0000919
8054722	IL1B	5.26	0.0000841
8095680	IL8	7.14	0.0000225

Supplementary Table 2

SGBS adipocytes on day 14 of adipogenic differentiation were treated with TRAIL (30 ng/ml) or vehicle. After 12 hours RNA was harvested and subjected to mRNA array analysis (Affymetrix, GeneChip Human Gene 1.0 ST Array). This table displays the genes that were differentially regulated by TRAIL in adipocytes. Downregulated genes are given in dark green, upregulated genes in red.

Probe Set	Gene	Fold-change TRAIL vs veh.	Parametric p-value
8069689	ADAMTS5	0.22	0.0000071
8148059	DEPTOR	0.27	0.0000135
8099524	LDB2	0.33	0.0000419
7962559	SLC38A4	0.35	0.0002488
8112731	F2RL2	0.40	0.003729
8051583	CYP1B1	0.41	0.0003349
7983630	FGF7	0.41	0.000522
7907160	ATP1B1	0.42	0.002565
7958262	TCP11L2	0.44	0.0051185
8095110	KIT	0.44	0.0000006
8104663	CDH6	0.44	0.0001977
8056257	FAP	0.44	0.0007663
8131844	GPNMB	0.44	0.0002771
8162388	OMD	0.45	0.0028756
8091537	IGSF10	0.45	0.0005825
8103166	SH3D19	0.45	0.0003881
8152703	FBXO32	0.45	0.0059712
7961580	LMO3	0.46	0.0065096
7921882	OLFML2B	0.46	0.0013561
8115397	C5orf4	0.47	0.0002006
7916493	PPAP2B	0.47	0.00009
7985317	KIAA1199	0.47	0.0009076
7919815	CTSK	0.47	0.0001969
8057677	SLC40A1	0.47	0.0004593
8152119	NCALD	0.48	0.0002567
7980908	FBLN5	0.48	0.0003805
8013341	MFAP4	0.48	0.0002866
8103415	FAM198B	0.49	0.0008911
7965403	LUM	0.51	0.000527
8090193	HEG1	0.52	0.0013675
7924996	C1orf198	0.52	0.0006266
8121601	FAM26E	0.53	0.0018403
8051028	NA	0.53	0.0459626
7965410	DCN	0.53	0.0015297
7966026	NUAK1	0.53	0.0029503
8104758	NPR3	0.55	0.0042965
8140971	SAMD9L	0.57	0.0023627
8002379	NA	1.56	0.0270812
7995258	ZNF267	1.59	0.0478315

7973067	PNP	1.85	0.0039919
7950983	CHORDC1	1.92	0.0238685
7954090	EMP1	1.96	0.0013212
8075635	TIMP3	2.04	0.0013826
7930008	NOLC1	2.08	0.0008748
8122265	TNFAIP3	2.13	0.0000053
8045688	TNFAIP6	2.13	0.000046
7943218	PANX1	2.13	0.0000973
8066513	SDC4	2.13	0.0001391
7992789	TNFRSF12A	2.17	0.0014676
8169603	LONRF3	2.22	0.0007681
8006433	CCL2	2.27	0.0002569
8021169	LIPG	2.27	0.0003352
8085914	SLC4A7	2.33	0.0001162
8116418	GFPT2	2.44	0.0004897
8138289	ETV1	2.50	0.0000652
7934979	ANKRD1	2.50	0.0000766
8095680	IL8	2.70	0.0000847
8131803	IL6	2.70	0.0005874
7958884	OAS1	2.78	0.0000759
8149865	EBF2	2.78	0.000077
8150076	DUSP4	2.86	0.0000228
8105267	ITGA2	2.94	0.0001832
8162531	NA	3.13	0.0000414
8091411	TM4SF1	3.13	0.0002427
8104901	IL7R	3.33	0.0004432
7995783	MT2A	3.57	0.0001384
8114797	SPRY4	3.70	0.0000041
7974366	PTGER2	3.70	0.0001796
8135069	SERPINE1	3.85	0.0000412
7906919	RGS4	3.85	0.0002209
7923547	CHI3L1	4.17	0.0000349
8141016	TFPI2	7.69	0.0000008
8021635	SERPINB2	12.05	0.0000064
8048864	CCL20	20.00	0.0002311

Supplementary Figure 1

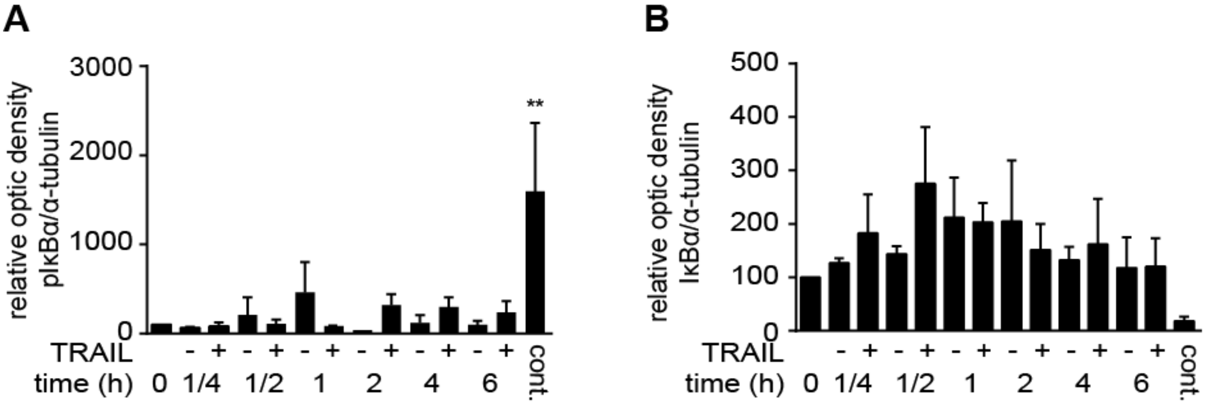

Supplementary Figure 1: TRAIL induces IκBα phosphorylation

SGBS adipocytes on day 14 of adipogenic differentiation were treated with TRAIL (30 ng/ml) or vehicle for the indicated times. Protein was isolated and the phosphorylation of IκBα as well as the expression of IκBα and α-tubulin were analyzed by Western blot. Relative levels of pIκBα, IκBα and α-tubulin were analyzed using Image J (https://imagej.nih.gov/ij). The levels of pIκBα (**A**) and IκBα (**B**) were first normalized to α-tubulin and then to the appropriate control (0 hours). Displayed are the means and SEM of 3 independent experiments. One-way ANOVA and Dunnett's multiple comparison were used to test for statistical significance. **; $p<0.01$.

Supplementary Figure 2

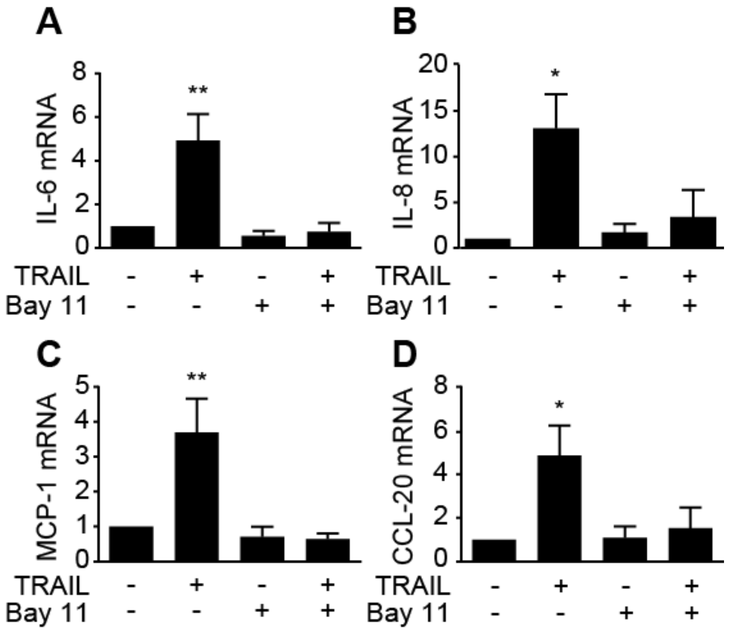

Supplementary Figure 2: Bay 11 inhibits the TRAIL-induced up-regulation of cytokines and chemokines in adipocytes

SGBS adipocytes on day 14 of adipogenic differentiation were treated with TRAIL (30 ng/ml) or vehicle in the absence or presence of the inhibitor Bay 11 (10 µM). After 6 hours, the expression of IL-6 (**A**), IL-8 (**B**), MCP-1 (**C**) and CCL-20 (**D**) was analyzed by qPCR. The mRNA levels were normalized to HPRT. Depicted is the means and SEM of 3 independent experiments. One-way ANOVA and Dunnett's multiple comparison were used to test for statistical significance. *; $p<0.05$, **; $p<0.01$.

Supplementary Figure 3

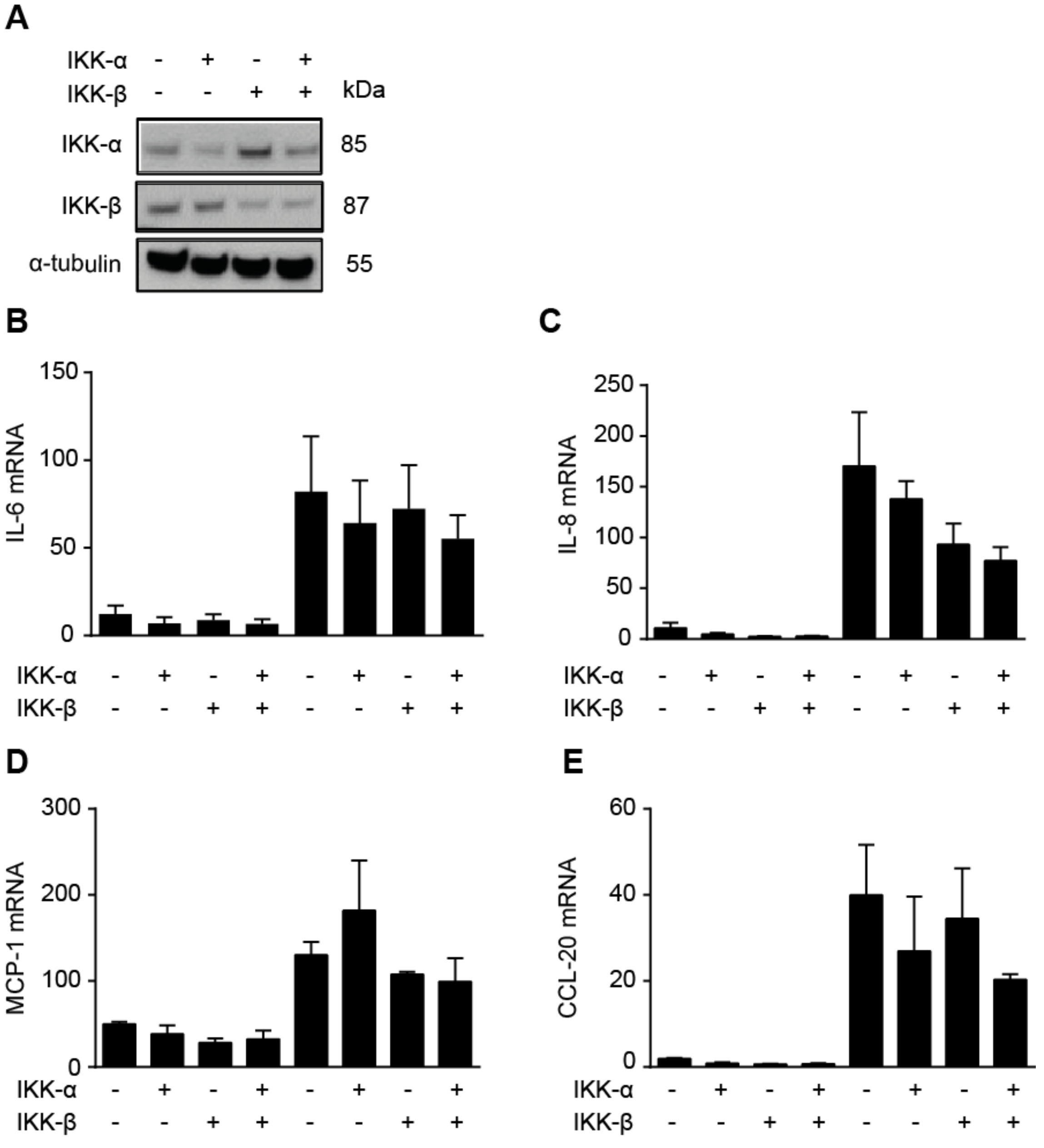

Supplementary Figure 3: Knockdown of IKK-α and IKK-β dampens the TRAIL-induced up-regulation of cytokines and chemokines in preadipocytes

SGBS predipocytes were transfected with siRNA constructs against IKK-α and IKK-β as published previously (IKK-α: 5'-GGA UGU UGG UGG AAA GAU A-3'; IKK-β: 5'-GGG AAC AAG ACC AGA GUU U-3'; Eurogentech, Lüttich, Belgium)(Jain et al. 2012, Neoplasia 14(3):178-89). Cells were seeded in 12-well plates with 50.000 cells per well and transfected the following day with 50 nM siRNA and 2.5 µl of Lipofectamin 2000 (Bio-Techne, Wiesbaden-Nordenstadt, Germany) per well. After 72 hours, the knockdown was controlled by Western blot (**A**). One representative blot out of three performed blots is presented. α-tubulin was used as a loading control. Also after 72 hours, the cells were treated with TRAIL (30 ng/ml) or vehicle. After another 6 hours, IL-6 (**B**), IL-8 (**C**), MCP-1 (**D**) and CCL-20 (**E**) expression was analyzed by qPCR. The mRNA levels were normalized to the gene HPRT. Depicted are the means and SEM of 4 independent experiments.

Supplementary Figure 4

Full-length blot of caspase-8, short exposure

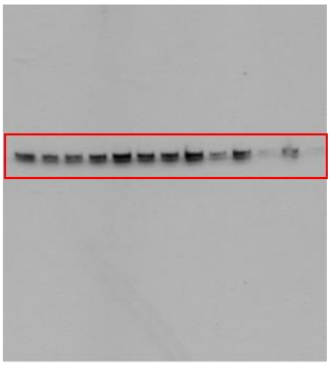

Full-length blot of caspase-3, short exposure

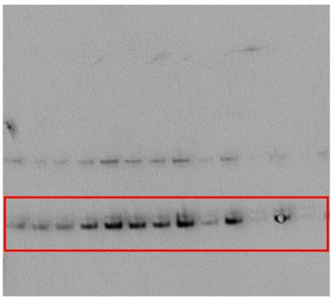

remaining caspase-8

Full-length blot of caspase-8, long exposure

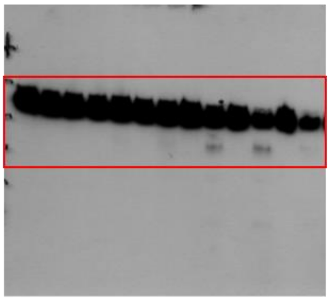

Full-length blot of caspase-3, long exposure

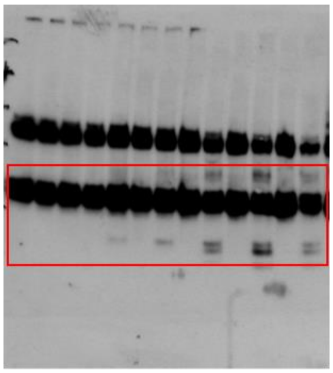

remaining caspase-8

Full-length blot of α-tubulin

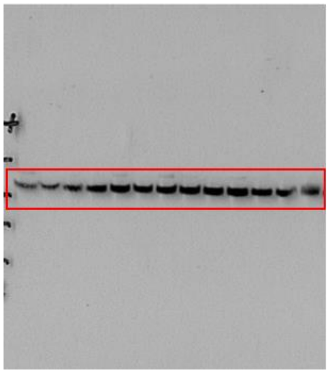

Supplementary Figure 4
The full-length blots for main Figure 4 are given. Bands shown in the main manuscript are marked in red. Membrane was first used for detection of caspase-8, then reprobed for detection of caspase-3.

Supplementary Figure 5

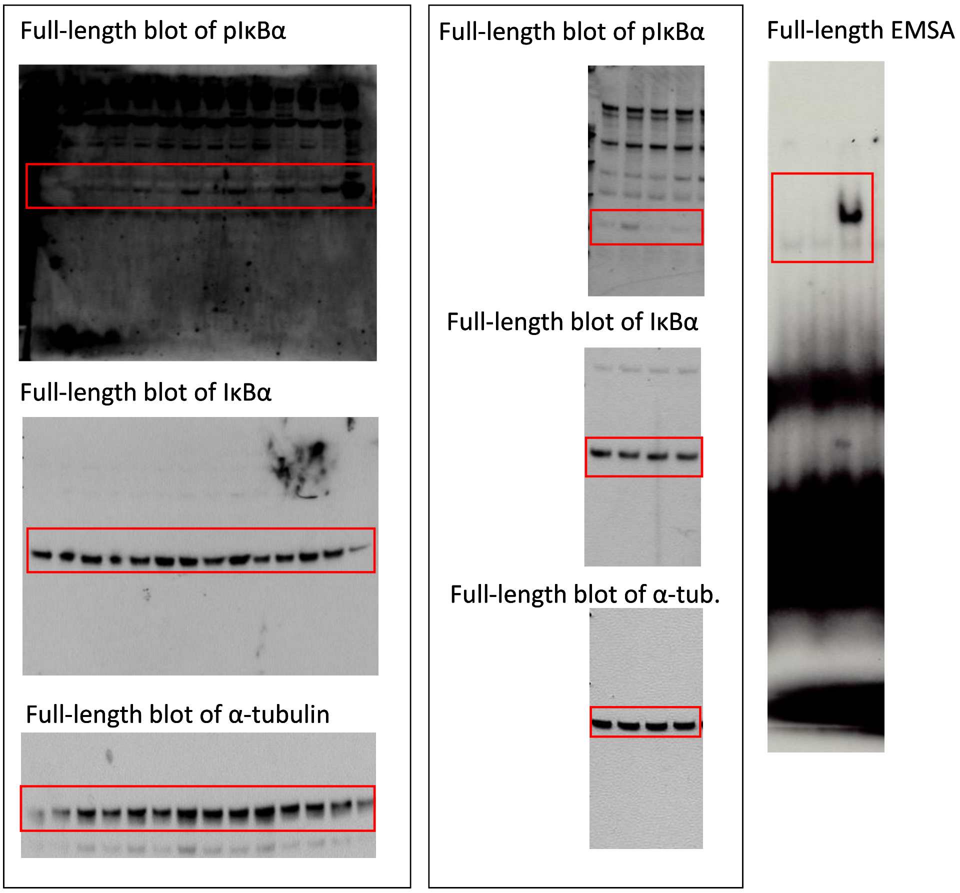

Supplementary Figure 5
The full-length blots for main Figure 5 are given. Bands shown in the main manuscript are marked in red. pIκBα antibody is known to produce unspecific bands.

Supplementary Figure 6

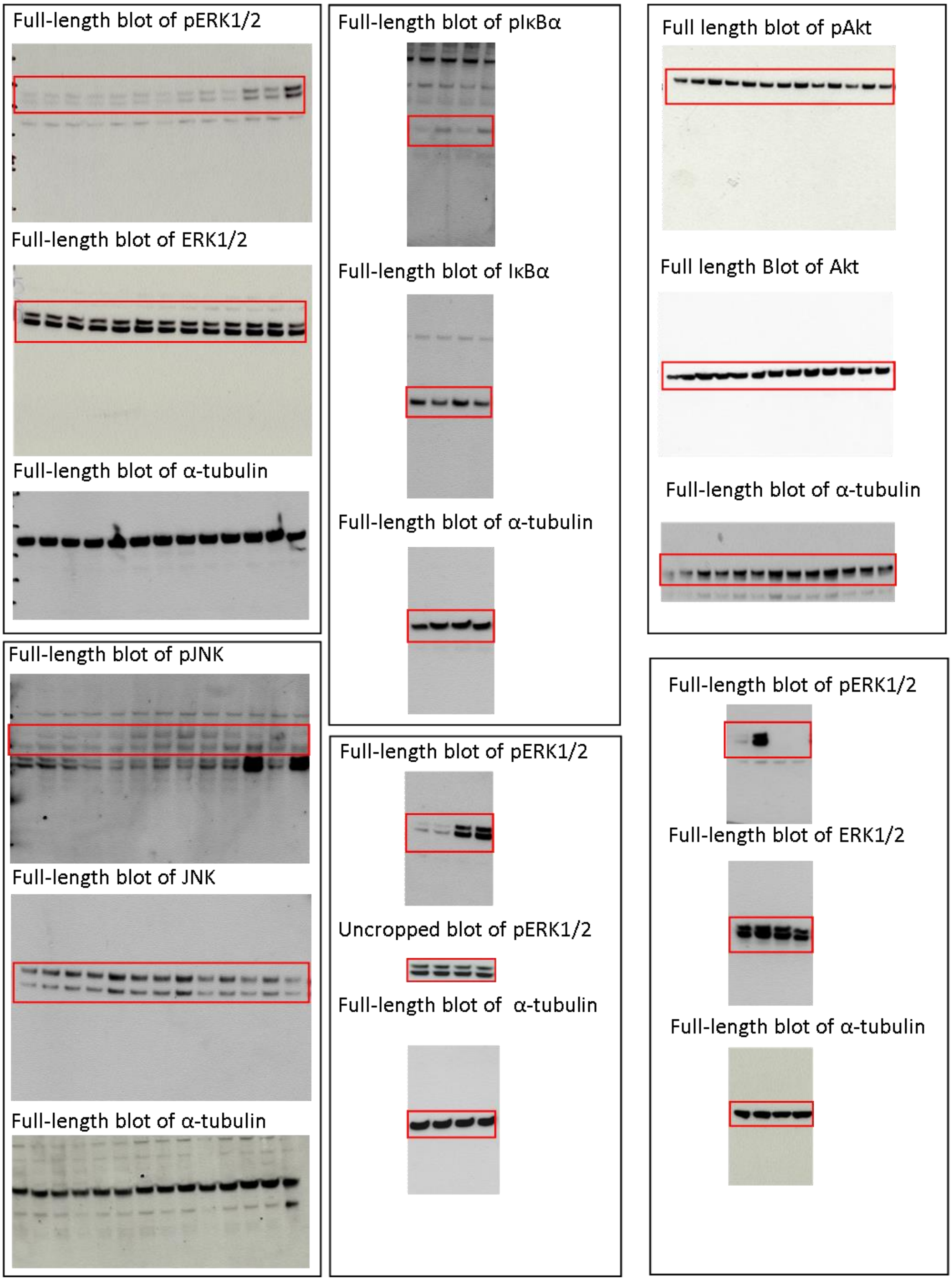

Supplementary Figure 6
The full-length blots for main Figure 6 are given. Bands shown in the main manuscript are marked in red. pJNK antibody showed cross-reactivity to pERK1/2.

Supplementary Figure 7

Full-length blot of IKK-β

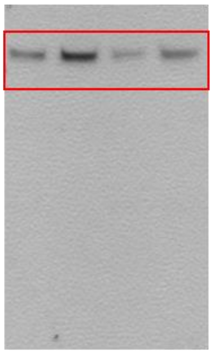

Full-length blot of IKK-α

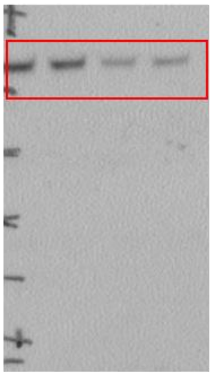

Full-length blot of α-tubulin

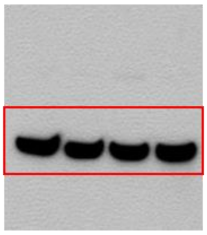

Supplementary Figure 7
The full-length blots for Supplementary Figure 3 are given. Used bands were marked red.
